# Lectures on Syphilis

**Published:** 1876-02

**Authors:** 


*Lectures on Syphilis, and on some forms of Local Disease affectiug principally the Organs of Generation.* By Henry Lee, Professor of Surgery in the Royal College of Surgeons, etc. Philadelphia: Henry C. Lea. 1875. Buffalo: T. Butler & Son.

The author says: "The principal object of the present work is to illustrate some of Hunter's doctrines which the lapse of time and the dissemination of more recent views have obscured or caused to be forgotten."

The author treats of the inoculability of syphilitic blood, the conditions under which the secretions of primary and secondary syphilitic manifestations may be inoculated naturally or artificially; the morbid processes produced by such inoculations; the modification of these processes in patients previously syphilitic; primary and secondary syphilitic diseases of the mucous membranes, and their liability to communicate constitutional syphilis; the essential differences of the morbid processes in which the constitutional and local forms of syphilis respectively have their origin; and the pathology and treatment of discharges from the prostrate gland, Cowper's glands, and the vesicutal seminales.

The work is worthy of attention as discussing some points in the etiology of syphilis, which do not, perhaps, receive sufficient consideration. Some of the lectures have appeared either as a whole or in abstract in the journals, but their publication in the present form will make them more attainable by the profession.

